# TILLING for allergen reduction and improvement of quality traits in peanut (*Arachis hypogaea *L.)

**DOI:** 10.1186/1471-2229-11-81

**Published:** 2011-05-12

**Authors:** Joseph E Knoll, M Laura Ramos, Yajuan Zeng, C Corley Holbrook, Marjorie Chow, Sixue Chen, Soheila Maleki, Anjanabha Bhattacharya, Peggy Ozias-Akins

**Affiliations:** 1 Department of Horticulture/NESPAL, University of Georgia-Tifton Campus, Tifton, GA 31793, USA; 2 USDA-ARS Crop Genetics and Breeding Research Unit, Tifton, GA 31793, USA; 3 Interdisciplinary Center for Biotechnology Research, University of Florida, Gainesville, FL 32611, USA; 4 USDA-ARS Southern Regional Research Center, New Orleans, LA 70124, USA

## Abstract

**Background:**

Allergic reactions to peanuts (*Arachis hypogaea *L.) can cause severe symptoms and in some cases can be fatal, but avoidance is difficult due to the prevalence of peanut-derived products in processed foods. One strategy of reducing the allergenicity of peanuts is to alter or eliminate the allergenic proteins through mutagenesis. Other seed quality traits could be improved by altering biosynthetic enzyme activities. Targeting Induced Local Lesions in Genomes (TILLING), a reverse-genetics approach, was used to identify mutations affecting seed traits in peanut.

**Results:**

Two similar copies of a major allergen gene, *Ara h 1*, have been identified in tetraploid peanut, one in each subgenome. The same situation has been shown for major allergen *Ara h 2*. Due to the challenge of discriminating between homeologous genes in allotetraploid peanut, nested PCR was employed, in which both gene copies were amplified using unlabeled primers. This was followed by a second PCR using gene-specific labeled primers, heteroduplex formation, CEL1 nuclease digestion, and electrophoretic detection of labeled fragments. Using ethyl methanesulfonate (EMS) as a mutagen, a mutation frequency of 1 SNP/967 kb (3,420 M_2 _individuals screened) was observed. The most significant mutations identified were a disrupted start codon in *Ara h 2.02 *and a premature stop codon in *Ara h 1.02*. Homozygous individuals were recovered in succeeding generations for each of these mutations, and elimination of Ara h 2.02 protein was confirmed. Several Ara h 1 protein isoforms were eliminated or reduced according to 2D gel analyses. TILLING also was used to identify mutations in fatty acid desaturase *AhFAD2 *(also present in two copies), a gene which controls the ratio of oleic to linoleic acid in the seed. A frameshift mutation was identified, resulting in truncation and inactivation of AhFAD2B protein. A mutation in *AhFAD2A *was predicted to restore function to the normally inactive enzyme.

**Conclusions:**

This work represents the first steps toward the goal of creating a peanut cultivar with reduced allergenicity. TILLING in peanut can be extended to virtually any gene, and could be used to modify other traits such as nutritional properties of the seed, as shown in this study.

## Background

Peanut (*Arachis hypogaea *L.) is an important source of oil and protein, and because of their nutritional benefits and versatility, peanuts and peanut-derived products are used extensively in processed foods. Unfortunately, reports of allergic reactions to peanuts are becoming increasingly common, and severe allergic reactions to peanuts can be fatal [1]. Avoidance is the best strategy to prevent allergic reactions, but due to the prevalence of peanuts in food products, avoidance can be difficult. Even food which does not specifically contain peanut products, but was processed on equipment also used for handling peanuts, can still contain significant amounts of allergens to trigger allergic response in some patients. Peanuts contain at least 11 potentially allergenic proteins, according to the International Union of Immunological Societies (IUIS) [2]. Knocking out the genes responsible for production of allergenic proteins would be one strategy for reducing the allergic potential of peanuts. However, many of these allergens are seed storage proteins which make up a considerable amount of the total seed protein. Major allergen Ara h 1, for example, makes up 12-16% of total seed protein, and Ara h 2 from 5.9-9.3% [3]. It is unknown how many of these proteins can be eliminated without sacrificing quality or viability, although Chu et al. [4] used transgenic silencing to eliminate Ara h 2 and Ara h 6 protein in peanut seeds, and observed no adverse effects on viability. Though such results are promising, there are many regulatory obstacles which must be overcome for a transgenic peanut to be used as food.

Another strategy is to use mutagenesis to knock out the allergen genes, or possibly to alter the sequences of major allergenic epitopes in those proteins. This can be accomplished though TILLING (Targeting Induced Local Lesions in Genomes), a technique in which a mutagenized population can be screened for individuals carrying mutations in any known gene of interest. TILLING is a PCR-based technique which relies on mismatch cleavage by CEL1 nuclease to identify single-nucleotide or small insertion/deletion mutations. TILLING was initially developed as a reverse-genetics tool in the model species *Arabidopsis thaliana*[5], but has since been applied to important crop species including rice (*Oryza sativa *L.) [6], maize (*Zea mays *L.) [7], and soybean (*Glycine max *(L.) Merr.) [8], to name just a few.

In a previous study we reported the genomic characterization of the major allergen gene *Ara h 2*[9]. Genes encoding the two isoforms, Ara h 2.01 and Ara h 2.02, are homeologous genes representing orthologs from diploid ancestors, most likely *A. duranensis *(A genome) and *A. ipaensis *(B genome). In this study we show that the major allergen *Ara h 1 *gene is also present in two copies, each belonging to separate subgenomes. Gene-specific primers were developed to identify mutations in each of the two *Ara h 1 *and two *Ara h 2 *genes through TILLING.

In addition to allergen reduction, seed oil composition is another quality trait in peanut that could be targeted using the TILLING approach. Monounsaturated fatty acids are less prone to oxidation than polyunsaturated fatty acids, and thus contribute to better flavor and longer storage life of peanut oil [10]. In addition, monounsaturated fatty acids are nutritionally desirable, and are believed to contribute to cardiovascular health. Linoleic acid (18:2) is a polyunsaturated fatty acid which typically makes up around 15-43% of peanut oil [11]. In developing seeds it is produced from the monounsaturated oleic acid (18:1) by the action of Δ^12 ^fatty acid desaturase (AhFAD2). Two homeologous *AhFAD2 *genes have been identified in peanut, one originating from each subgenome, designated *AhFAD2A *and *AhFAD2B*[12]. Reduction in the activity of AhFAD2 increases the ratio of oleic to linoleic acid, but only one functioning allele is required to confer a normal oleate phenotype [13]. Mutations in each of the *AhFAD2 *genes were also identified using TILLING.

## Results

### Determination of Gene Copy Numbers, and Gene-Specific Amplification

Prior to TILLING in a polyploid such as peanut it is necessary to determine the copy number and perform the molecular characterization of any gene of interest, because most genes exist in multiple copies. Co-amplification of multiple homologous sequences would likely result in an excessive number of fragments on TILLING gels, and difficulty in identification of mutations. Also when a mutation is identified, it is necessary to know which gene copy has been altered. In peanut, which is an allotetraploid, genes encoding the two isoforms of Ara h 2 are homeologous, representing orthologs from diploid ancestors [9]. The open reading frames of these two genes are highly similar, with the major difference being an in-frame insertion of 36 bp in *Ara h 2.02*, resulting in an insertion of 12 amino acids containing an extra copy of the sequence DPYSPS, a known allergenic IgE-binding epitope [14,15]. Gene-specific primer pairs yielded amplicons of 1,280 bp for *Ara h 2.01 *and 1,227 bp for *Ara h 2.02 *(Table 1). Each primer pair amplified only one band of expected size from the A- or B-subgenome, and also from the putative progenitors *A. duranensis *and *A. ipaensis*, respectively [16]. Furthermore, the specific amplification was confirmed by sequence analysis (data not shown).

**Table 1:** Summary of amplicon sizes and frequencies of mutations identified by TILLING in two different EMS treatments

	Amplicon	Screened	**0.4% EMS/12 hr**.	**1.2% EMS/4.5 hr**.	Total
Gene	bp		No. of Mutations:		
*Ara h 1.01*	2211	2011	2	2	4
*Ara h 1.02*	1666	1466	1	0	1
*Ara h 2.01*	1278	1078	7	2	9
*Ara h 2.02*	1226	1026	2	3	5
*Ah FAD2A*	1228	1028	5	0	5
*Ah FAD2B*	1221	1021	3	0	3
**Total:**	8830	7630	20	7	27

		**Plants Screened:**	2441	979	3420
		**kb/SNP:**	931	1067	966

For number of bp screened, 200 bp is subtracted to adjust for the 100-bp regions at the top and bottom of TILLING gel images that are difficult to analyze.

**Figure 1:**
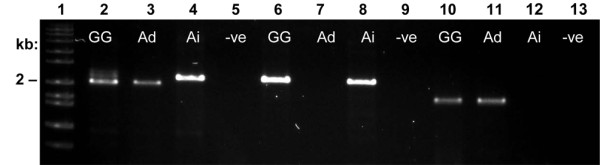
**PCR amplification of *Ara h 1 *isoforms on 1% agarose gel**. Lane 1: DNA size standard. Lanes 2-5: primers 1306/1307 amplify both isoforms of *Ara h 1*. Lanes 6-9: primers 1306/1308 amplify only *Ara h 1.01*. Lanes 10-13: primers 1306/1309 amplify only *Ara h 1.02*. GG = *A. hypogaea *cv. Georgia Green, Ad = *A. duranensis *(A genome), Ai = *A. ipaensis *(B genome), -ve = negative control.

**Figure 2:**
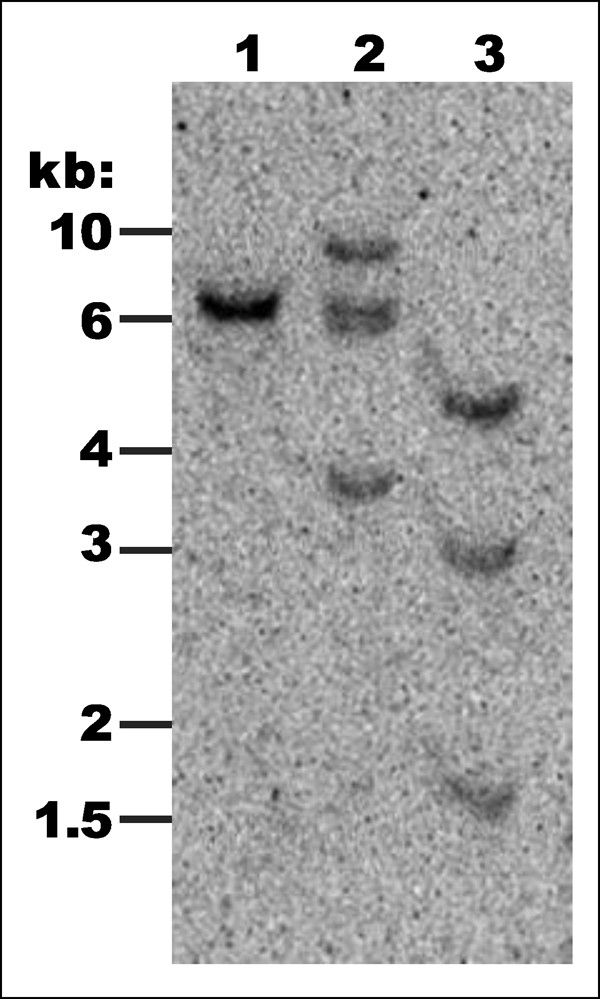
**Southern blot analysis of *Ara h 1 *in *A. hypogaea *cv. Georgia Green**. The blot was probed with a full-length genomic fragment of *Ara h 1.01*, which was PCR-amplified from a plasmid, then labeled with ^32^P. Lane 1: Genomic DNA digested with *Hin*dIII (no sites within either gene). Lane 2: Genomic DNA digested with *Eco*RI (one site in each gene). Lane 3: Genomic DNA digested with *Ase*I (two adjacent cut sites in *Ara h 1.01 *(B-genome), but none in *Ara h 1.02 *(A-genome)).

Prior to designing PCR primers for *Ara h 1*, two genomic clones of *Ara h 1 *were found in GenBank. The first accession [GenBank: AF432231] was reported by Viquez et al. [17] and is identical to the cDNA sequence of accession L34402 whose encoded protein is designated Ara h 1.0101 by IUIS [2] (isoform Ara h 1.01). A second genomic clone [GenBank: AY581852] was reported by Li et al. [18] and is nearly identical to accession L38853 whose protein is referred to by Chassaigne et al. [19] as isoform 2. For clarity we will refer to this isoform as Ara h 1.02 even though this is not an official IUIS designation. PCR amplification using primers 1306 and 1307 (Table 2) produced two PCR products appearing as a doublet on agarose gel (2,241 bp for *Ara h 1.01*, and 2,031 bp for *Ara h 1.02*; Figure 1). Amplicons from gene-specific PCR were 2,211 bp for *Ara h 1.01 *and 1,666 bp for *Ara h 1.02 *(Figure 1; Table 1). Analysis of *Ara h 1 *PCR products from *A. hypogaea *and its diploid progenitors showed the presence of both genes in *A. hypogaea*, but only one copy in each diploid. The primer pair specific to *Ara h 1.01 *(1306/1308; Table 2) amplified only in *A. hypogaea *and *A. ipaensis *(B genome), while the primer pair specific to *Ara h 1.02 *(1306/1309; Table 2) amplified only in *A. hypogaea *and *A. duranensis *(A genome; Figure 1). Using the known sequence information, Southern blot analysis of genomic DNA from *A. hypogaea *was carried out to confirm that no additional copies of *Ara h 1 *are present in the peanut genome. Genomic DNA digested with *Hin*dIII, which has no cut sites within either gene, yielded two nearly overlapping fragments of approximately 6.5 kb each when probed with a full-length *Ara h 1.01 *probe (PCR product of primers 1306/1308). DNA was also digested with *Eco*RI, which has one cut site in each copy of *Ara h 1*. Southern blot analysis revealed four fragments, two from each homeolog, as expected. Lastly, the DNA was cut with *Ase*I, which cuts *Ara h 1.01 *(two adjacent cut sites within the second intron), but not *Ara h 1.02*. As expected, three fragments were produced (Figure 2). *Eco*RI-digested plasmids carrying either *Ara h 1.01 *or *Ara h 1.02 *were also loaded as controls; the probe recognized both copies of the gene (data not shown).

**Table 2:** PCR primers used in this study.

**Primer no**.	Description	Sequence (5'-3')
813	5' *Ara h 2*	GGAGTGAAAAAGAGAAGAGAATA
817	3' *Ara h 2*	TCAAGATGGTTACAACTCTGCAGCAACA
815	5' *Ara h 2.01*	CGATTTACTCATGTACAATTAACAATAGAT
816	5' *Ara h 2.02*	ATCACCTTAAATTTATACATATTTTCGG
371	3' *Ara h 2*	CAGCAACAAAACATAGACAACGCC
1306	5' *Ara h 1*	GAGCAATGAGAGGGAGGGTT
1307	3' *Ara h 1*	CCTCCTTGGTTTTCCTCCTC
1308	3' *Ara h 1.01*	TTCTCAGGAGACTCTTTCTCAGG
1309	3' *Ara h 1.02*	CCTCCTCTTCTTCCCACTCTTG
1048	3' *AhFAD2*	CTCTGACTATGCATCAG
1055	5' *AhFAD2A*	GATTACTGATTATTGACTT
1101	5' *AhFAD2B*	CAGAACCATTAGCTTTG
1458	3' *AhFAD2*	CAGAACTTGTTCTTGTACCAATAAACACC
1459	5' *AhFAD2B*	TCAGAACCATTAGCTTTGTAGTAGTGC
1460	5' *AhFAD2A*	GATTACTGATTATTGACTTGCTTTGTAG

Another target for TILLING, the Δ^12^-fatty acid desaturase gene *AhFAD2 *has been characterized in studies by Jung et al. [12], López et al. [20], and Patel et al. [21]. This gene is also present in two copies, one in each subgenome of *A. hypogaea*. The gene sequences are highly conserved between the two, except for an insertion of 19 bp in *AhFAD2A *(or a deletion in *AhFAD2B*), 80 bp upstream of the start codon. Gene-specific primer sequences utilizing this indel produce amplicons nearly identical in size: 1,228 bp for *AhFAD2A *and 1,221 bp for *AhFAD2B *(Table 1).

### Peanut TILLING Populations and Mutation Frequencies

Several populations were created using ethyl methanesulfonate (EMS) and one with diethylsulfate (DES). The concentration of mutagen and time of treatment were selected from preliminary experiments that gave 30%-50% seed germination. From the DES-treated M_2 _population, 352 plants were screened for all six genes, and no mutations were detected. Two EMS mutagenesis treatments were tested in this study, 1.2% for 4.5 h and 0.4% for 12 h. A total of 3,420 EMS-treated M_2 _plants were screened, each for all six genes (7,630 bp/plant; Table 1). Twenty-seven SNPs were detected and confirmed. The overall mutation frequency for EMS was 1 SNP/967 kb. For 1.2% EMS at 4.5 h, the mutation rate was 1 SNP/1,067 kb (979 plants). The mutation frequency for 0.4% EMS for 12 h was slightly higher at 1 SNP/931 kb (2,441 plants), although this difference probably is not significant. Most of the nucleotide changes were G to A or C to T, as expected for EMS-induced transitions. Several unusual mutations were found in *AhFAD2A *and *AhFAD2B*, which may not be the result of the EMS treatment (Table 3). If that is the case, then the average mutation frequency would be 1 SNP/1186 kb.

**Table 3:** Mutations identified by TILLING in this study

**Treatment:**0.4% EMS for 12 hr.				
**Gene**	**Nucleotide Change**	**Predicted AA Change**	**Population**	**Plant ID**

*Ara h 2.01*	C145 → T	L49 → F	05	20-6
*Ara h 2.01*	G164 → A	R55 → H	05	37-4
*Ara h 2.01*	G192 → A	silent	05	37-4
*Ara h 2.01*	G186 → A	silent	07G	78-4
*Ara h 2.01*	C80 → T	silent	07G	90-4
*Ara h 2.01*	G357 → A	silent	07JKEMS1	65
*Ara h 2.01*	G186 → A	silent	08GH	250
*Ara h 2.02*	G185→ A	R62 → Q	07G	89-5
*Ara h 2.02*	G3 → A	disrupted start codon	08GH	2
*Ara h 1.01*	C1392→ T	R333 → W	07G	95-1
*Ara h 1.01*	C586 → T	silent	07JKEMS1	99
*Ara h 1.02*	C304 → T	R102 → Stop	07JKEMS1	133
*AhFAD2A*	A448 → G	N150 → D	05	4-3
*AhFAD2A*	A448 → G	N150 → D	05	55-4
*AhFAD2A*	A448 → G	N150 → D	05	138-10
*AhFAD2A*	C718 → T	silent	07G	113-5
*AhFAD2A*	C761 → T	P254 → L	07JKEMS1	72
*AhFAD2B*	A442 insertion	frameshift	05	69-8
*AhFAD2B*	A442 insertion	frameshift	07G	81-4
*AhFAD2B*	C566 → T	silent	07JKEMS1	2

**Treatment:**1.2% EMS for 4.5 hr.				

**Gene**	**Nucleotide Change**	**Predicted AA Change**	**Population**	**Plant ID**
*Ara h 2.01*	G243 → A	A82 → T	06EF	13-6
*Ara h 2.01*	G192 → A	silent	06LREMS1	8-4
*Ara h 2.02*	G208 → A	D70 → N	06EF	23-7
*Ara h 2.02*	G208 → A	D70 → N	06EF	26-1
*Ara h 2.02*	G -315 → A	upstream, probably silent	06EF	62-6
*Ara h 1.01*	C1609 → T	P405 → L	06EF	53-3
*Ara h 1.01*	G1704 → A	E437 → K	06EF	56-3

### *Ara h 2 *Mutations

**Figure 3:**
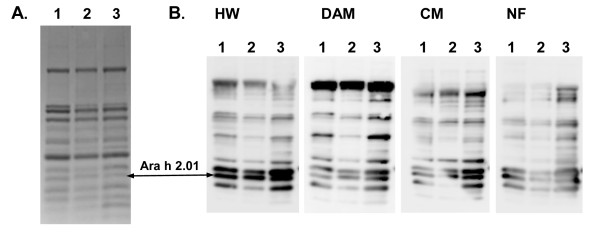
**IgE binding analysis of seed protein extracts from M_4 _generation of Ara h 2.01 mutant lines 20-6 and 37-4**. A - Equal amount of total protein from seeds of wild type (Georgia Green; Lane 1), mutant line 37-4 (Lane 2), and mutant line 20-6 (Lane 3) loaded on SDS-PAGE stained with Coomassie blue. B - IgE inmunoblot performed with serum from patients with peanut hypersensitivity (HW, DAM, CM, and NF). Lane numbers are the same as in panel 4A.

In total, nine SNPs were identified in *Ara h 2.01*, and five in *Ara h 2.02*. The first two amino-acid changes identified were in *Ara h 2.01 *in lines 20-6 (L 49 F) and 37-4 (R 55 H; Table 3). Line 37-4 actually had two nucleotide changes in this gene, but one of them was silent. These two mutations were confirmed in the M_3 _and M_4 _generations using TILLING. DNA from M_3 _or M_4 _individuals was analyzed both alone and mixed with wild type DNA. Homozygotes were identified when SNPs were detected in mixed samples but not in the corresponding unmixed samples. Homozygous mutants allowed the testing of IgE binding on the altered proteins from seed extracts. Total protein extracts from homozygous M_4 _lines of 20-6 and 37-4 were normalized for loading equal amounts of Ara h 2.01, as measured by anti-Ara h 2 chicken polyclonal antibody, and were tested for binding to serum from four patients with peanut hypersensitivity (HW, DAM, CM, and NF). The IgE-immunoblot showed no differences between the native Ara h 2.01 present in the peanut cultivar Georgia Green (GG) [22] and the Ara h 2.01 allelic variants detected by TILLING in lines 20-6 and 37-4 (Figure 3). Although the mutations were generated in cultivar Tifrunner [23] there is no difference between the Ara h 2.01 proteins of these two cultivars.

Four more silent mutations were found in *Ara h 2.01*, one of which is identical to the silent mutation in line 37-4. One other amino acid change (A 82 T) was also identified in *Ara h 2.01*. Three amino acid changes were identified in *Ara h 2.02*, but two of them (D 70 N) are identical (Table 3). This change occurs in the second DPYSPS motif, which is a known allergenic epitope [14,15]. The third amino acid change (R 62 Q) also lies within an allergenic epitope, just before the first DPYSPS motif (Additional File 1). Because homozygous seed has not yet been recovered, the ability of these mutant proteins to bind IgE has not yet been tested, although these look to be promising candidates for reduced allergenicity of Ara h 2.02. A G to A mutation was also found 315 bp upstream of the start codon of *Ara h 2.02*; however, it does not appear to be located within any important promoter elements.

**Figure 4:**
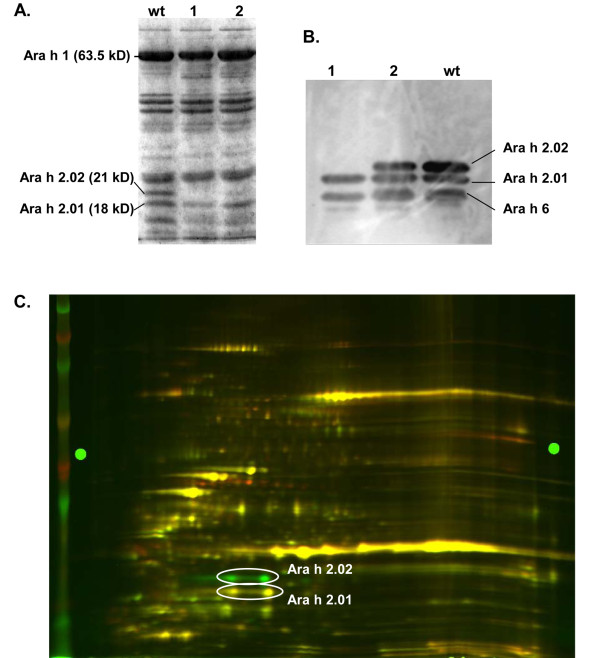
**Analysis of seed protein extracts from Ara h 2.02 knockout mutant**. A - Coomassie blue stained SDS-PAGE of seed protein extracts, with equal amounts of total protein loaded in each lane. Lane wt: wild type (Tifrunner). Lane 1: homozygous mutant. Lane 2: heterozygote. B - Western blot of seed protein extracts using anti-Ara h 2 antibodies, which recognize both isoforms of the allergen. Antibodies also recognize Ara h 6. Lane wt: wild type (Tifrunner). Lane 1: homozygous mutant. Lane 2: heterozygote. C - 2D DIGE analysis of seed protein extracts from wild-type (Tifrunner) labeled with Cy3 (green) and Ara h 2.02 knockout mutant labeled with Cy2 (red). The white box denotes the four spots representing Ara h 2 isoforms.

Lastly, a G to A transition was identified in the start codon of *Ara h 2.02*. A downstream ATG is out of frame, and so a protein knockout was expected. Two M_3 _seeds were recovered, a small chip was taken from each for protein analysis, and the seeds were planted. Both seeds grew into phenotypically normal plants. SDS-PAGE analysis of the seed protein extracts confirmed that one of the seeds was indeed missing the 21 kD band which represents the Ara h 2.02 protein [9], and was thus homozygous for the mutation (Figure 4A). The other seed appeared to have a reduced quantity of Ara h 2.02; DNA sequence analysis (data not shown) confirmed that this plant was a heterozygote. Western blot analysis (Figure 4B) also confirmed the absence of Ara h 2.02 protein in the homozygous mutant. Further analysis with 2-D difference gel electrophoresis (2-D DIGE) confirmed that both of the Ara h 2.02 isoforms, shown to differ by a two amino acid truncation at the carboxy terminus [24], were missing in the homozygous mutant line (Figure 4C).

### *Ara h 1 *Mutations

**Figure 5:**
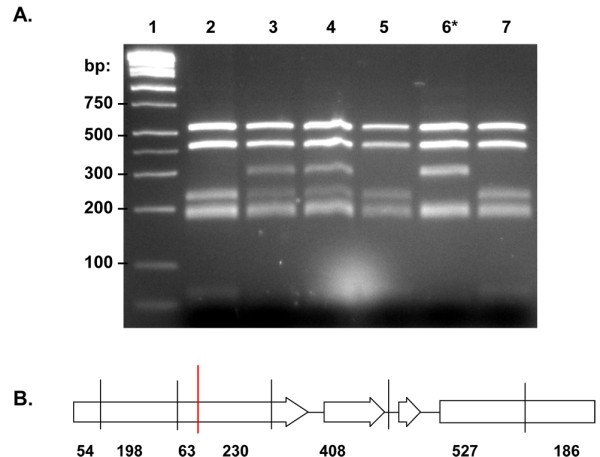
**Identification of *Ara h 1.02 *truncation mutant by CAPS marker analysis**. A - Primers 1306/1309 were used to amplify *Ara h 1.02 *from M_3 _individuals. PCR products were cut with *Bsl*I and then separated on 2% agarose gel. Lane 1: DNA size marker. Lane 2: wild-type control (Tifrunner). Lanes 3-7: individual M_3 _plants. The 293 bp fragment indicates presence of the mutant allele. The homozygous mutant (Lane 6) lacks the 230 bp fragment. B - Diagram of the amplified fragment of *Ara h 1.02*. Vertical lines represent *Bsl*I cut sites. The cut site denoted in red is eliminated by the mutation.

In the longest amplicon, *Ara h 1.01 *(2,211 bp), signals from both IRDye channels sometimes were not visible on Li-Cor gels due to background and fragment length, but SNPs identified from single-channel signals were later verified by sequencing. Four mutations have been confirmed in *Ara h 1.01 *(Table 1). One of these, a C to T transition at bp position 593, is silent, but the other three are predicted to induce amino acid changes: R 333 W, P 405 L, and E 437 K (Table 3; Additional File 2). The arginine to tryptophan change at position 333 lies within epitope 12 [25]. Only one mutation was confirmed in *Ara h 1.02*; a premature stop codon is produced at bp position 304 by a C to T mutation. This is expected to result in a truncated protein of 102 amino acids (Line 133; Additional File 2). All four of these non-silent mutations have been confirmed in the M_3 _generation by TILLING. A CAPS (cleaved amplified polymorphic sequence) marker was developed to detect the *Ara h 1.02 *truncation mutant in succeeding generations. The wild-type amplicon contains six *Bsl*I sites, one of which is deleted in the mutant. This marker was used to identify a homozygous mutant in the M_4 _generation (Figure 5).

**Figure 6:**
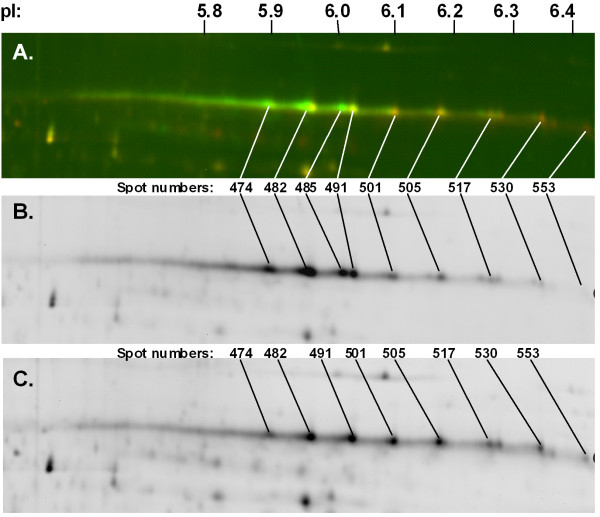
**2D DIGE analysis of Ara h 1.02 truncation mutant**. Protein extracted from seeds of homozygous wild-type (Tifrunner) was labeled with Cy3 (green), and seed protein from Ara h 1.02 truncation mutant was labeled with Cy5 (red). Labeled proteins were separated by 2-D DIGE with a pI range of 5.3-6.5. Region of 2-D gel where most Ara h 1 protein separates is shown in detail. A - Two-color image. Wild-type protein is green; mutant protein is red. B - Single-color image of wild-type protein only. C - Single-color image of mutant protein.

Both Ara h 1 proteins appear as a thick band of approximately 63.5 kD on SDS-PAGE [26]. Although the two genes encode proteins of slightly different sizes, we were unable to resolve both of them with one-dimensional electrophoresis. Thus, 2D SDS-PAGE and 2D-DIGE were attempted to confirm the absence of the protein in seeds of the homozygous *Ara h 1.02 *truncation mutant. From the 2-D PAGE and 2-D Western blot (Additional File 3) it was not possible to resolve only two distinct Ara h 1 isoforms, an expected result based on published 2-D gel analyses for Ara h 1 [19]. Multiple post-translational protein modifications (i.e. various cleavage products or glycosylation) are produced from the two isoforms of Ara h 1. However, there was a definite difference in the relative Cy3 (wild-type) and Cy5 (mutant) signal intensities for the group of spots in the pI range of 5.9-6.4 representing Ara h 1. From these data it is not possible to conclude that the Ara h 1.02 isoform has been completely eliminated. However, quantitative analysis of the 2-D DIGE mutant and wild-type gels showed that the intensities of three pI 5.9-6.0 spots representing Ara h 1 (Figure 6A, spots 474, 482, 485) were reduced 2.4-2.6-fold in the mutant, but others with a higher pI appeared to increase (1.5-3.5-foldTable 4), although these isoforms were less abundant than the lower pI isoforms in both wild-type and mutant. Also, spots 482 and 485/491 which appear as doublets in the wild-type (Figure 6B) appear as single spots in the mutant (Figure 6C), suggesting that several protein products have indeed been eliminated in the mutant.

**Table 4:** Change in abundance of Ara h 1 protein isoforms in homozygous truncation mutant, relative to wild-type

**Spot No**.	pI	Mass (kD)	Max Volume	Volume Ratio	Abundance
474	5.90	56,559	1,596,575	-2.57	Decreased
482	5.96	56,281	2,763,712	-2.40	Decreased
485	6.02	56,147	1,360,494	-2.67	Decreased
491	6.04	56,052	1,480,757	-1.27	Similar
501	6.10	55,805	639,866	1.55	Increased
505	6.18	55,745	827,950	1.53	Increased
517	6.28	55,488	338,574	1.51	Increased
530	6.35	55,220	270,914	3.00	Increased
553	6.42	54,463	131,587	3.57	Increased

(Spot numbers correspond to Figure 6.)

### *AhFAD2 *Mutations

One silent mutation was found in each of *AhFAD2A *and *AhFAD2B*, and one predicted amino acid change (P 254 L) was found in *AhFAD2A*. All three of these mutations were C to T transitions, typical for EMS-induced mutations. Several mutations were also identified in these genes which were not typical: an A-insertion, observed twice in *AhFAD2B*, and three identical A to G mutations in *AhFAD2A *(Table 3). These are unusual for EMS-induced mutations, but it is perhaps the location and frequency of these mutations which is most intriguing. The A-insertion in *AhFAD2B *occurs 442 bp after the start codon, causing a frameshift, and likely resulting in a truncated protein due to a premature stop codon (line 81-4; Additional File 4). This mutation was identified in two different M_2 _plants in our TILLING populations. Using a CAPS marker [27], this mutation has been shown to be stably inherited in the M_3 _generation derived from one of our TILLING mutants (data not shown). In *AhFAD2A*, three different M_2 _plants were found to contain the same mutation, an A to G transition at 448 bp after the start codon. This is predicted to change the amino acid at position 150 from asparagine to aspartic acid (line 4-3; Additional File 4).

## Discussion

In TILLING populations of diploids such as sorghum (*Sorghum bicolor *(L.) Moench) [28] and *Lotus japonicus*[29], phenotypic mutants were frequently observed. In contrast, very few phenotypic mutations were observed in field or greenhouse-grown M_2 _peanut plants in this study, most likely due to genetic buffering caused by polyploidy, similar to that observed in TILLING populations of tetraploid and hexaploid wheat (*Triticum aestivum *L.) [30]. In EMS-mutagenized hexaploid wheat, a mutation frequency of 1 SNP/24 kb has been reported, and 1 SNP/40 kb was reported in tetraploid wheat [30]. The mutation rate observed in this study on peanut is much lower than that reported for wheat and lower than *Arabidopsis *(1 SNP/~300 kb [4]), or most legumes including soybean (1 SNP/140-550 kb depending on treatment [8]), and pea (*Pisum sativum *L.; 1 SNP/669 kb [31]; 1 SNP/200 kb [32]). It is similar to or higher than that in some populations of barley (*Hordeum vulgare *L.; 1 SNP/2500 kb [33], 1 SNP/1000 kb [34]) and rice (1 SNP/1000 kb [35]). As with barley and rice, mutation density potentially could be improved by using alternate genotypes, treatment conditions, or choice of mutagens [6,36]. No mutations were detected in the DES-mutagenized population, even though this chemical was used to recover a high oleic acid mutant of peanut [37]. In the present study, an incubation time of 4.5 h at a concentration of 0.25% was substantially different from that used by Moore [37] (15 min at 1.5%). With the longer incubation time of 4.5 h, no germination occurred at a concentration greater than 0.5%.

The IgE-immunoblot showed no differences between the wild-type Ara h 2.01 and the Ara h 2.01 allelic variants detected by TILLING in lines 20-6 and 37-4 (Figure 3), despite the fact that both of these changes affect known IgE epitopes [14,15]. Although a reduction in IgE binding was not detected with these two allelic variants, it has been shown that a small change in this protein can indeed have this desired effect. In a recent study Ramos et al. [38] identified a naturally occurring variant (a serine to threonine change at position 73) in an accession of *A. duranensis *that showed 56-99% reduction in IgE binding compared to wild-type Ara h 2.01. The arginine to tryptophan change at position 333 in Ara h 1.01 lies within epitope 12 [25]. Although it is unlikely that this residue is critical for IgE binding [25], and the other two amino acid changes do not reside within known epitopes, the possibility of reduced IgE affinity cannot be completely ruled out until these proteins are tested.

The *Ara h 1.01 *and *Ara h 1.02 *genes code for proteins with predicted sizes of 71.3 and 70.3 kD, respectively, but the mature proteins extracted from seeds appear as a single 63.5 kD band on SDS-PAGE [26]. The N-terminal amino acid sequence of the purified proteins does not match the predicted N-terminal sequence; rather it is located 78 or 84 amino acids downstream, depending on the isoform [39,40]. These first 78 or 84 amino acids, along with an included 25 amino acid signal peptide, are cleaved off during post-translational processing. The 53 or 59 amino acid cleaved peptides contain six of the seven cysteines found in Ara h 1 isoforms [40] and three of the allergenic epitopes [41], and are hypothesized to form disulfide bridges conferring a stable conformation similar to plant antifungal peptides [40]. In our *Ara h 1.02 *truncation mutant, the truncation occurs downstream of the cleavage site potentially leaving the cleaved peptide intact. It remains to be seen whether the cleavage product is still produced and stable in seeds of the mutant.

A previously described mutant allele of *AhFAD2B *contains an A-insertion 442 bp after the start codon, causing a frameshift, and likely results in a truncated protein due to a premature stop codon [20]. This mutant allele has been reported previously in multiple independently derived cultivars which have a high oleic to linoleic acid ratio (high O/L), most likely due to the inactivity of AhFAD2B [27]. The same mutation was identified in two different M_2 _plants in our TILLING populations. It is possible that this mutant allele is present at a low frequency in the source seed for the TILLING population, although these seed were produced before extensive breeding for the high O/L trait was initiated in the USDA-ARS program. Furthermore, independent generation of this mutant allele has been reported in China and the U.S. [27]. Even more surprising, three different M_2 _plants were found to contain a reversion to the wild-type allele of *AhFAD2A*, an A to G transition at 448 bp after the start codon, whereas the TILLING population parent, 'Tifrunner', possesses the mutant allele. This reversion is predicted to change the amino acid at position 150 from asparagine to aspartic acid and restore functionality to the desaturase enzyme. In most runner-type peanut cultivars, the AhFAD2A protein is presumed to be inactive due to the presence of the asparagine residue at position 150 [42]. The aspartic acid residue is likely an important component of the active site of the enzyme and is highly conserved among fatty acid desaturases from other plants, including *A. duranensis*, from which *AhFAD2A *likely is derived [13]. Based on a survey of the mini-core of the U.S. peanut germplasm collection, Chu et al. [42] found that the aspartic acid residue also appears to be conserved among Spanish and Valencia market types of peanut, but the inactive allele was found to be common (75%) among Virginia and Runner market-types. In our three independent TILLING mutants, the asparagine has been mutated back to aspartic acid, most likely restoring the function of AhFAD2A. In a recombinant AhFAD2A protein with the aspartic acid restored at position 150 by site-directed mutagenesis, Bruner et al. [43] showed that its full function is indeed restored. Both the frequency and the nature of these two mutations are atypical of mutations induced by EMS, including the other mutations observed in this study. It is unclear whether these mutations are due to the EMS treatment, outcrossing, or genetic impurity in the starting seed, but the latter appears to be the most likely explanation. If that is the case, then assessment of genetic purity at specific loci may be another use for mismatch-based mutation detection.

## Conclusions

These experiments represent the initial steps toward the development of a hypoallergenic peanut. Because genetic variation for allergens is limited in cultivated peanut, mutagenesis is necessary to generate variation. We have shown that TILLING is a useful technique for screening mutagenized populations of peanut for induced changes in allergen genes. When multiple seed storage proteins with reduced IgE binding are identified, or more knockout mutations are found, the next step will be a concerted breeding effort to combine these mutant alleles into one plant. TILLING, CAPS markers, or a more efficient SNP assay can be used as tools to track the inheritance of these alleles in the breeding process. TILLING in peanut can be extended to virtually any gene, and could be used to assist in the modification of other traits such as disease resistance, stress tolerance, early maturity, or as shown in this study, nutritional properties of the seed.

## Methods

### Southern Blot Analysis of *Ara h 1*

DNA for Southern blot analysis was isolated from young leaves of peanut (*Arachis hypogaea *L.) cv. Georgia Green [22] using the DEAE-cellulose-based technique of Sharma et al. [44]. Twenty micrograms of purified genomic DNA was digested overnight with *Ase*I, *Eco*RI, or *Hin*dIII, and was then loaded on a 0.7% agarose gel and electrophoresed in TBE buffer at 45 V for approximately nine hours. *Eco*RI-digested pCR-4 TOPO plasmids (Invitrogen, Carlsbad, CA) carrying either *Ara h 1.01 *or *Ara h 1.02 *(clones derived from PCR products using primer pairs 1306/1308 and 1306/1309, respectively; Table 2) were also loaded in adjacent lanes as positive controls. The DNA was transferred to Genescreen Plus nylon membrane (Perkin-Elmer, Boston, MA) overnight using the alkaline transfer method [45]. The membrane was probed with a full-length genomic fragment of *Ara h 1.01*, which was PCR-amplified from a plasmid carrying the fragment. The probe was labeled with α^32^P-dCTP using the Random Primed DNA Labelling Kit (Roche, Indianapolis, IN). Unincorporated label was removed using Sephadex G-50 (Sigma, Saint Louis, MO). Hybridization and washing conditions were as described by Sambrook and Russell [45]. The final wash was carried out at 65°C for 15 min. in 0.5 × SSC buffer (75 mM NaCl, 7.5 mM sodium citrate, pH 7.0) with 0.1% SDS. The blot was visualized by exposure to a Storage Phosphor Screen (Amersham Biosciences, Piscataway, NJ) which was then scanned using a Storm 840 imaging system (Amersham Biosciences).

### Mutant Peanut Populations

Ethyl methanesulfonate (EMS) or diethylsulfate (DES) treatments were used to induce mutations in the peanut cultivar 'Tifrunner' [23]. Seeds were imbibed in tap water for 10-12 hours. The tap water was then replaced with aqueous solution of mutagen. Three mutagen treatments were tested: 0.4% EMS for 12 h, 1.2% EMS for 4.5 h, or 0.25% DES for 4.5 h. Seeds were soaked in the mutagen solution in 2L Fernbach flasks on a rotary shaker, and were then washed three times in deionized water. (Washes were collected for disposal). The seeds were then rinsed in running water overnight. The M_1 _seeds were planted in the field, and one pod was harvested from each plant to generate an M_2 _population. M_2 _seeds were planted in either the field or greenhouse, and M_3 _seed was harvested from them to create permanent TILLING populations.

The entire population will not be distributed because of limited seed availability, although screening for specific mutant genes and distribution of individual lines is possible.

### DNA Isolation and Quantification for TILLING

Young leaf tissue was collected from individual M_2 _plants, frozen using liquid nitrogen, and either stored at -80°C or lyophilized directly in 96-well collection plates. It was then ground into powder by vortexing with three to four 3-mm stainless-steel grinding balls in 2-ml flat-bottom microcentrifuge tubes, or using a GenoGrinder 2000 (OPS Diagnostics LLC, Bridgeview, NJ), set at 500 strokes/min for 20 sec (liquid nitrogen-frozen tissue), or 1 min (lyophilized tissue). Genomic DNA was extracted using the DNeasy 96 Plant Kit (Qiagen Inc. USA, Valencia, CA) according to the manufacturer's instructions. The DNA was quantified by fluorometry using either PicoGreen (Invitrogen, Carlsbad, CA) or Hoechst 33258 dye in a FluoroCount (Packard/Perkin-Elmer, Waltham, MA) microplate reader. Samples of purified DNA were also run on agarose gel to verify quality. Individual DNA samples were diluted to a working concentration of 5 ng/μl. Individual DNA samples were then four-fold pooled in 96-well format. For verification of individual mutants, genomic DNA from 'Tifrunner' was used as the control.

### Primer Design and PCR

Since *Ara h 2 *genes are small and without introns, differences in the upstream regions of these two genes were used to design gene-specific primers for TILLING (Primers 815 and 816). Based on the available sequence information in GenBank, primers 1306 and 1307 were designed to amplify both copies of *Ara h 1*. Indels near the 3' end of the open reading frame allowed us to design gene-specific primers 1308 (*Ara h 1.01*) and 1309 (*Ara h 1.02*). Primer sequences 1055 (*AhFAD2A*) and 1101 (*AhFAD2B*) utilize the indel 80 bp upstream of the start codon to amplify one specific gene copy. These primers are identical to primers aF19 and bF19 used by Patel et al. [21]. For amplification with IRDye-labeled primers, longer oligos are preferred, so primers 1458, 1459, and 1460 were designed. All primer sequences used in this study are shown in Table 2.

Because peanut DNA is highly complex, a first round of unlabeled PCR was used to increase the concentration of target sequences for subsequent labeled PCR. Based on available sequence information and suitability of priming sites, primers for the first round of PCR were designed to amplify both copies of *Ara h 2*, both copies of *Ara h 1*, or one specific copy of *AhFAD2*. The first PCR was carried out in a 25 μl final volume containing 10 ng gDNA, 0.5 U JumpStart *Taq *DNA Polymerase in 1 × PCR Buffer (Sigma, Saint Louis, MO), 0.2 mM each dATP, dCTP, dGTP and dTTP, and 0.2 μM each forward and reverse primers, under the following conditions: 94°C for 1 min; followed by 8 cycles at 94°C for 35 sec, 58°C for 35 sec (-1°C/cycle), 72°C for 100 sec. The touchdown cycles were followed by 30 cycles of 94°C for 35 sec, 50°C for 35 sec, 72°C for 100 sec, with a final extension of 72°C for 7 min. Reactions were conducted using either a Gene Amp 9700 (Applied Biosystems, Carlsbad, CA) or a PTC-200 (MJ Research, Waltham, MA) thermal cycler.

An aliquot (2 μl) from a 1:40 dilution of the first PCR product was used as input for a second round of PCR, carried out in 10 μl final volume with 0.2 mM each dNTP, 0.25 U ExTaq HS DNA Polymerase (TaKaRa Bio Inc, Shiga, Japan) with IRDye-labeled primers (MWG Biotech, Huntsville, AL), designed to specifically amplify one gene copy. Labeled and unlabeled primers (100 μM stocks) were mixed into a cocktail in a ratio of 3 parts IRD-700-labeled 5' primer: 2 parts unlabeled 5' primer: 4 parts IRD-800-labeled 3' primer: 1 part unlabeled 3' primer. Concentrations of primer cocktail, PCR buffer, and MgCl_2 _were optimized for each individual gene. Touchdown PCR was conducted in a PTC-200 thermal cycler (MJ Research, Waltham, MA) as follows: denaturation at 95°C for 2 min followed by 6 cycles of 94°C for 30 sec, 58°C for 30 sec (-1°C/cycle), temperature ramp +0.5°C/sec to 72°C for 80 sec; then 45 cycles of 94°C for 30 sec, 52°C for 30 sec with a temperature ramp +0.5°C/sec to 72°C for 80 sec. This was followed by a final extension at 72°C for 7 min. PCR was immediately followed by the heteroduplex formation step: denaturation at 99°C for 10 min, 70 cycles of reannealing at 70°C for 20 sec, decreasing 0.3°C/cycle, with a final hold at 4°C.

### Preparation of Celery Juice Extract (CEL1 Nuclease)

Celery juice extract (CJE), containing CEL1 nuclease, was prepared following the purification protocol from Till et al. [46] with minor modifications. The endonuclease activity and the concentration were tested using a plasmid nicking assay as follows: 200 ng of circular plasmid were incubated with 10 μl of CJE dilution in 1 × CELI Buffer (10 mM MgSO_4_, 10 mM HEPES, 10 mM KCl, 0.02% Triton X-100, 0.002% bovine serum albumin) in 20 μl final volume. After incubation at 45°C for 15 min, the sample was placed on ice and 10 μl of 0.15 M EDTA was added to stop the reaction. The digestion products were analyzed on 1% agarose gel. The activity of the CJE was compared with that of Surveyor Nuclease (Transgenomic, Omaha, NE) on a known mutant, detected previously by EcoTILLING [38,47].

### Mutation Screening

After PCR amplification, samples (5 μl from the second PCR) were digested in 1 × CEL1 Buffer with 0.03-0.06 μl CJE in 10 μl total volume, incubated for 15 min at 45°C as described by Till et al. [46]. To stop the reaction 5 μl of 0.15 M EDTA was added per sample, while keeping the samples on ice. The samples were cleaned using Sephadex G-50 (Sigma, Saint Louis, MO), uniformly loaded in 96-well MultiScreen-HV filter plates using a 45-μl MultiScreen Column Loader (Millipore, Billerica, MA) following the manufacturer's instructions. The samples were collected in a catch plate, transferred to a 96-well PCR plate, and dried in an ISS110 Speed Vac centrifugal evaporator (Thermo Savant, Milford, MA). The dried samples were resuspended in 8 μl of formamide loading buffer (37% formamide, 3.75 mM EDTA pH 8, 0.0075% bromophenol blue), and then heated to 80°C for 7 min, and then to 92°C for 2 min [30]. Samples could then be stored in the dark at 4°C for several days until analysis. Samples (0.8 μl) were loaded on 6.5% polyacrylamide gel in 1 × TBE and electrophoresed at 1500 V, 40 mA, 30 W, at 45°C on a Li-Cor 4300 DNA Analyzer (Li-Cor Biosciences, Lincoln, NE). Images were visually analyzed for the presence of cleavage products using Adobe Photoshop (Adobe Systems, Inc, San Jose, CA) and GelBuddy [48]. Putative mutations were identified by fragments appearing in both the 700 and 800 channels, with sizes adding up to that of the full-length PCR product. Because the DNA was pooled four-fold for initial screening, each of the four individuals was then screened against wild type (Tifrunner) to identify the mutant.

Mutations were confirmed by cloning PCR-amplified genes or gene fragments into the pCR4-TOPO cloning vector using the TOPO-TA Cloning Kit for Sequencing (Invitrogen, Carlsbad, CA) according to the manufacturer's instructions, followed by sequencing with M13 forward and reverse primers. Mutation frequency was estimated as the total number of confirmed mutations divided by the total number of base pairs screened [(amplicon size - 200 bp) × individuals screened)]. For each amplicon, 200 bp is subtracted to adjust for the 100-bp regions at the top and bottom of TILLING gel images that are difficult to analyze [5].

### SDS-PAGE, Western Blots, and IgE-Binding Analysis

Seed protein extraction was carried out as described by Koppelman et al. [3]. Protein quantification was performed using the Bio-Rad Protein Assay kit (Bio-Rad, Hercules, CA) and bovine serum albumin (BSA) as the standard. Ten micrograms of total protein were loaded per lane and separated in 12% SDS-PAGE gels. The proteins were then electroblotted at 30 V overnight to PVDF membranes, 0.2 μm (Bio-Rad). The membranes were blocked with 5% non-fat dry milk in PBS-T (137 mM NaCl, 2.7 mM KCl, 1.4 mM KH_2_PO_4_, 4.3 mM Na_2_HPO_4_, 0.1% Tween-20) at room temperature for 1 h with gentle agitation. For Western blot analysis, after blocking the membrane was probed with chicken anti-Ara h 2 or anti-Ara h 1 (1:8,000 dilution) primary antibodies for 1 h followed by secondary anti-chicken antibody-horseradish peroxidase (HRP; 1:100,000; Sigma, Saint Louis, MO) in PBS-T with 2% non-fat dry milk for 1 h. For IgE binding analysis, sera were obtained from four individuals (HW, DAM, CM, and NF) documented as allergic to peanuts with either a positive food challenge or a convincing history of peanut allergy. Sera from allergic individuals were collected at the University of Arkansas for Medical Sciences, Little Rock, AR, approved and in accordance with the rules and regulations of the institutional review board. Blots incubated with a 1:20 dilution of the four sera were allowed to hybridize overnight at 4°C. All membranes were washed three times with PBS-T for 5 min after each antibody incubation. ECL Plus detection reagents (Amersham Biosciences) were used for chemifluorescent detection, followed by scanning on a Storm 840 imaging system (Amersham Biosciences). For IgE binding analysis, relative quantification of Ara h 2.01 protein was performed by densitometry with Quantity One software (Bio-Rad) and all samples were normalized to the amount of Ara h 2.01/10 μg of total protein in wild-type. Further IgE immunoblot analyses were performed using 100 ng of Ara h 2.01-normalized total protein.

### 2-D PAGE and DIGE

Suspected homozygous knockout mutants of Ara h 2.02 and Ara h 1.02 were subjected to two-dimensional protein analyses using difference gel electrophoresis (2-D DIGE) essentially according to Chu et al. [4]. Labeled protein samples for analysis of 17-19 kD Ara h 2 (wild-type - Cy3; mutant - Cy2) were resolved using p*I *3-10 immobilized pH gradient (IPG) strips. For analysis of ~65 kD Ara h 1, 50 μg each of Cy3-labeled wild-type, Cy5-labeled mutant, and Cy2-labeled mixture of wild-type and mutant protein extracts were mixed and focused in a 24-cm pH 5.3-6.5 IPG strip at 10,000 V for 121 kVhr before running the second dimension in a 10% Tris-glycine SDS-PAGE gel. Labeled spots were detected with a Typhoon 9400 Variable Mode Imager (GE Healthcare) and digital image analysis was conducted with SameSpot Progenesis (Nonlinear Dynamics).

## Authors' contributions

JEK performed Southern blot analysis for *Ara h 1*, screened and identified mutants, and drafted the manuscript. MLR developed the TILLING protocols used in this study, prepared celery juice extract, and screened and identified mutants. AB assisted in screening and identifying mutants. POA was the lead investigator, generated the mutagenized populations, and also drafted the manuscript. YZ, MC, and SC performed 2D PAGE and DIGE on the protein extracts from knockout mutants. SM performed IgE binding analysis on mutant Ara h 2.01 proteins. CCH assisted in generating, advancing, and maintaining the mutagenized populations. All authors have read and approved the manuscript.

## Authors' information

Current addresses:

JEK - USDA-ARS Crop Genetics and Breeding Research Unit, Tifton, GA 31793, USA

MLR - NIDERA S.A., Departamento de Biotecnologia, Venado Tuerto, Santa Fe CP2600, Argentina

AB - Bench Biotechnology, Vapi, Gujarat, India

## Supplementary Material

Additional file 1 **Sequence alignment of Ara h 2.01 and Ara h 2.02 wild-type proteins and predicted proteins from Ara h 2 mutants identified by TILLING**. WT indicates wild-type protein sequence. Mutant ID numbers are indicated in parentheses. Click here for file

Additional file 2 **Sequence alignment of Ara h 1.01 and Ara h 1.02 wild-type proteins and predicted proteins from Ara h 1 mutants identified by TILLING**. WT indicates wild-type protein sequence. Mutant ID numbers are indicated in parentheses. Click here for file

Additional file 3 **2D PAGE and Western blot of Ara h 1.02 truncation mutant**. A - Sypro Ruby stained PVDF blots of seed protein extracts (1.5 mg) from wild-type (Tifrunner) and homozygous Ara h 1.02 truncation mutant. Proteins were first focused in pH 5.3 to 6.5 IPG strips, then separated in 10% polyacrylamide Tris-glycine gels before transblotting to PVDF membrane. B - Western blot of membranes in panel 7A using chicken anti-Ara h 1 antibody (primary) followed by anti-chicken-HRP conjugate (secondary), and visualized by fluorescence. Click here for file

Additional file 4 **Sequence alignment of AhFAD2A and AhFAD2B wild-type proteins and predicted proteins from AhFAD2 mutants identified by TILLING**. WT indicates wild-type protein sequence. Mutant ID numbers are indicated in parentheses. Click here for file
