# Supplementary material for: TILLING for allergen reduction and improvement of quality traits in peanut (Arachis hypogaea L.)
Source: BMC Plant Biol. 2011 May 12;11:81. doi: 10.1186/1471-2229-11-81 (PMC3113929; doi:10.1186/1471-2229-11-81)
Supplement: Additional file 1 — Sequence alignment of Ara h 2.01 and Ara h 2.02 wild-type proteins and predicted proteins from Ara h 2 mutants identified by TILLING. WT indicates wild-type protein sequence. Mutant ID numbers are indicated in parentheses. [file 1471-2229-11-81-S1.PDF]

```
                                                                                               Section 1
                         (1) 1        10        20        30        40        50           63
   Ara h 2.01 (wt)       (1) MAKLTILVALALFLLAAHASARQQWELQGDRRCQSQLERANLRPCEQHLMQKIQRDEDSYERD
   Ara h 2.01 (13-6)     (1) MAKLTILVALALFLLAAHASARQQWELQGDRRCQSQLERANLRPCEQHLMQKIQRDEDSYERD
   Ara h 2.01 (20-6)     (1) MAKLTILVALALFLLAAHASARQQWELQGDRRCQSQLERANLRPCEQHFMQKIQRDEDSYERD
   Ara h 2.01 (37-4)     (1) MAKLTILVALALFLLAAHASARQQWELQGDRRCQSQLERANLRPCEQHLMQKIQHDEDSYERD
   Ara h 2.02 (wt)       (1) MAKLTILVALALFLLAAHASARQQWELQGDRRCQSQLERANLRPCEQHLMQKIQRDEDSYGRD
   Ara h 2.02 (23-7)     (1) MAKLTILVALALFLLAAHASARQQWELQGDRRCQSQLERANLRPCEQHLMQKIQRDEDSYGRD
   Ara h 2.02 (26-1)     (1) MAKLTILVALALFLLAAHASARQQWELQGDRRCQSQLERANLRPCEQHLMQKIQRDEDSYGRD
   Ara h 2.02 (89-5)     (1) MAKLTILVALALFLLAAHASARQQWELQGDRRCQSQLERANLRPCEQHLMQKIQRDEDSYGQD
                                                                                               Section 2
                        (64) 64    70        80        90        100       110             126
   Ara h 2.01 (wt)      (64) PYSPSQ------------DPYSPSPYDRRGAGSSQHQERCCNELNEFENNQRCMCEALQQIME
   Ara h 2.01 (13-6)    (64) PYSPSQ------------DPYSPSPYDRRGTGSSQHQERCCNELNEFENNQRCMCEALQQIME
   Ara h 2.01 (20-6)    (64) PYSPSQ------------DPYSPSPYDRRGAGSSQHQERCCNELNEFENNQRCMCEALQQIME
   Ara h 2.01 (37-4)    (64) PYSPSQ------------DPYSPSPYDRRGAGSSQHQERCCNELNEFENNQRCMCEALQQIME
   Ara h 2.02 (wt)      (64) PYSPSQDPYSPSQDPDRRDPYSPSPYDRRGAGSSQHQERCCNELNEFENNQRCMCEALQQIME
   Ara h 2.02 (23-7)    (64) PYSPSQNPYSPSQDPDRRDPYSPSPYDRRGAGSSQHQERCCNELNEFENNQRCMCEALQQIME
   Ara h 2.02 (26-1)    (64) PYSPSQNPYSPSQDPDRRDPYSPSPYDRRGAGSSQHQERCCNELNEFENNQRCMCEALQQIME
   Ara h 2.02 (89-5)    (64) PYSPSQDPYSPSQDPDRRDPYSPSPYDRRGAGSSQHQERCCNELNEFENNQRCMCEALQQIME
                                                                                               Section 3
                       (127) 127          140       150       160         173
   Ara h 2.01 (wt)     (115) NQSDRLQGRQQEQQFKRELRNLPQQCGLRAPQRCDLDVESGGRDRY-
   Ara h 2.01 (13-6)   (115) NQSDRLQGRQQEQQFKRELRNLPQQCGLRAPQRCDLDVESGGRDRY-
   Ara h 2.01 (20-6)   (115) NQSDRLQGRQQEQQFKRELRNLPQQCGLRAPQRCDLDVESGGRDRY-
   Ara h 2.01 (37-4)   (115) NQSDRLQGRQQEQQFKRELRNLPQQCGLRAPQRCDLDVESGGRDRY-
   Ara h 2.02 (wt)     (127) NQSDRLQGRQQEQQFKRELRNLPQQCGLRAPQRCDLEVESGGRDRY-
   Ara h 2.02 (23-7)   (127) NQSDRLQGRQQEQQFKRELRNLPQQCGLRAPQRCDLEVESGGRDRY-
   Ara h 2.02 (26-1)   (127) NQSDRLQGRQQEQQFKRELRNLPQQCGLRAPQRCDLEVESGGRDRY-
   Ara h 2.02 (89-5)   (127) NQSDRLQGRQQEQQFKRELRNLPQQCGLRAPQRCDLEVESGGRDRY-
```
