# Supplementary material for: TILLING for allergen reduction and improvement of quality traits in peanut (Arachis hypogaea L.)
Source: BMC Plant Biol. 2011 May 12;11:81. doi: 10.1186/1471-2229-11-81 (PMC3113929; doi:10.1186/1471-2229-11-81)
Supplement: Additional file 2 — Sequence alignment of Ara h 1.01 and Ara h 1.02 wild-type proteins and predicted proteins from Ara h 1 mutants identified by TILLING. WT indicates wild-type protein sequence. Mutant ID numbers are indicated in parentheses. [file 1471-2229-11-81-S2.PDF]

Section 1

```
                        (1) 1        10        20        30        40        50           63
       Ara h 1.01 (wt)  (1) MRGRVSPLMLLLGILVLASVSATHAKSSPYQKKTENPCAQRCLQSCQQEPDDLKQKACESRCT
     Ara h 1.01 (53-3)  (1) MRGRVSPLMLLLGILVLASVSATHAKSSPYQKKTENPCAQRCLQSCQQEPDDLKQKACESRCT
     Ara h 1.01 (56-3)  (1) MRGRVSPLMLLLGILVLASVSATHAKSSPYQKKTENPCAQRCLQSCQQEPDDLKQKACESRCT
     Ara h 1.01 (95-1)  (1) MRGRVSPLMLLLGILVLASVSATHAKSSPYQKKTENPCAQRCLQSCQQEPDDLKQKACESRCT
       Ara h 1.02 (wt)  (1) MRGRVSPLMLLLGILVLASVSATQAK-SPY-RKTENPCAQRCLQSCQQEPDDLKQKACESRCT
      Ara h 1.02 (133)  (1) MRGRVSPLMLLLGILVLASVSATQAK-SPY-RKTENPCAQRCLQSCQQEPDDLKQKACESRCT
```

Section 2

```
                       (64) 64    70        80        90        100       110            126
       Ara h 1.01 (wt) (64) KLEYDPRCVYDPRGHTGTTNQRSPPGERTRGRQPGDYDDDRRQPRREEGGRWGPAGPRERERE
     Ara h 1.01 (53-3) (64) KLEYDPRCVYDPRGHTGTTNQRSPPGERTRGRQPGDYDDDRRQPRREEGGRWGPAGPRERERE
     Ara h 1.01 (56-3) (64) KLEYDPRCVYDPRGHTGTTNQRSPPGERTRGRQPGDYDDDRRQPRREEGGRWGPAGPRERERE
     Ara h 1.01 (95-1) (64) KLEYDPRCVYDPRGHTGTTNQRSPPGERTRGRQPGDYDDDRRQPRREEGGRWGPAGPRERERE
       Ara h 1.02 (wt) (62) KLEYDPRCVYD----TGATNQRHPPGERTRGRQPGDYDDDRRQPRREEGGRWGPAEPRERERE
      Ara h 1.02 (133) (62) KLEYDPRCVYD----TGATNQRHPPGERTRGRQPGDYDDDRRQP-------------------
```

Section 3

```
                      (127) 127          140       150       160       170                189
      Ara h 1.01 (wt) (127) EDWRQPREDWRRPSHQQPRKIRPEGREGEQEWGTPGSHVREETSRNNPFYFPSRRFSTRYGNQ
    Ara h 1.01 (53-3) (127) EDWRQPREDWRRPSHQQPRKIRPEGREGEQEWGTPGSHVREETSRNNPFYFPSRRFSTRYGNQ
    Ara h 1.01 (56-3) (127) EDWRQPREDWRRPSHQQPRKIRPEGREGEQEWGTPGSHVREETSRNNPFYFPSRRFSTRYGNQ
    Ara h 1.01 (95-1) (127) EDWRQPREDWRRPSHQQPRKIRPEGREGEQEWGTPGSHVREETSRNNPFYFPSRRFSTRYGNQ
      Ara h 1.02 (wt) (121) EDWRQPREDWRRPSHQQPRKIRPEGREGEQEWGTPGSEVREETSRNNPFYFPSRRFSTRYGNQ
     Ara h 1.02 (133) (102) ---------------------------------------------------------------
```

Section 4

```
                      (190) 190       200       210       220       230       240        252
      Ara h 1.01 (wt) (190) NGRIRVLQRFDQRSRQFQNLQNHRIVQIEAKPNTLVLPKHADADNILVIQQGQATVTVANGNN
    Ara h 1.01 (53-3) (190) NGRIRVLQRFDQRSRQFQNLQNHRIVQIEAKPNTLVLPKHADADNILVIQQGQATVTVANGNN
    Ara h 1.01 (56-3) (190) NGRIRVLQRFDQRSRQFQNLQNHRIVQIEAKPNTLVLPKHADADNILVIQQGQATVTVANGNN
    Ara h 1.01 (95-1) (190) NGRIRVLQRFDQRSRQFQNLQNHRIVQIEAKPNTLVLPKHADADNILVIQQGQATVTVANGNN
      Ara h 1.02 (wt) (184) NGRIRVLQRFDQRSKQFQNLQNHRIVQIEARPNTLVLPKHADADNILVIQQGQATVTVANGNN
     Ara h 1.02 (133) (102) ---------------------------------------------------------------
```

Section 5

```
                      (253) 253    260       270       280       290       300            315
      Ara h 1.01 (wt) (253) RKSFNLDEGHALRIPSGFISYILNRHDNQNLRVAKISMPVNTPGQFEDFFPASSRDQSSYLQG
    Ara h 1.01 (53-3) (253) RKSFNLDEGHALRIPSGFISYILNRHDNQNLRVAKISMPVNTPGQFEDFFPASSRDQSSYLQG
    Ara h 1.01 (56-3) (253) RKSFNLDEGHALRIPSGFISYILNRHDNQNLRVAKISMPVNTPGQFEDFFPASSRDQSSYLQG
    Ara h 1.01 (95-1) (253) RKSFNLDEGHALRIPSGFISYILNRHDNQNLRVAKISMPVNTPGQFEDFFPASSRDQSSYLQG
      Ara h 1.02 (wt) (247) RKSFNLDEGHALRIPSGFISYILNRHDNQNLRVAKISMPVNTPGQFEDFFPASSRDQSSYLQG
     Ara h 1.02 (133) (102) ---------------------------------------------------------------
```

```
                                                                        Section 6
                (316) 316           330       340       350       360             378
     Ara h 1.01 (wt) (316) FSRNTLEAAFNAEFNEIRRVLLEENAGGEQEERGQRRWSTRSSENNEGVIVKVSKEHVEELTK
   Ara h 1.01 (53-3) (316) FSRNTLEAAFNAEFNEIRRVLLEENAGGEQEERGQRRWSTRSSENNEGVIVKVSKEHVEELTK
   Ara h 1.01 (56-3) (316) FSRNTLEAAFNAEFNEIRRVLLEENAGGEQEERGQRRWSTRSSENNEGVIVKVSKEHVEELTK
   Ara h 1.01 (95-1) (316) FSRNTLEAAFNAEFNEIWRVLLEENAGGEQEERGQRRWSTRSSENNEGVIVKVSKEHVEELTK
     Ara h 1.02 (wt) (310) FSRNTLEAAFNAEFNEIRRVLLEENAGGEQEERGQRRRSTRSSDN-EGVIVKVSKEHVQELTK
    Ara h 1.02 (133) (102) ---------------------------------------------------------------
                                                                        Section 7
                (379) 379        390       400       410       420       430      441
     Ara h 1.01 (wt) (379) HAKSVSKKGSEEEGDITNPINLREGEPDLSNNFGKLFEVKPDKKNPQLQDLDMMLTCVEIKEG
   Ara h 1.01 (53-3) (379) HAKSVSKKGSEEEGDITNPINLREGELDLSNNFGKLFEVKPDKKNPQLQDLDMMLTCVEIKEG
   Ara h 1.01 (56-3) (379) HAKSVSKKGSEEEGDITNPINLREGEPDLSNNFGKLFEVKPDKKNPQLQDLDMMLTCVKIKEG
   Ara h 1.01 (95-1) (379) HAKSVSKKGSEEEGDITNPINLREGEPDLSNNFGKLFEVKPDKKNPQLQDLDMMLTCVEIKEG
     Ara h 1.02 (wt) (372) HAKSVSKKGSEEE-DITNPINLRDGEPDLSNNFGRLFEVKPDKKNPQLQDLDMMLTCVEIKEG
    Ara h 1.02 (133) (102) ---------------------------------------------------------------
                                                                        Section 8
                (442) 442     450       460       470       480       490        504
     Ara h 1.01 (wt) (442) ALMLPHFNSKAMVIVVVNKGTGNLELVAVRKEQQQRGRRE---EEEDEDEEEEGSNREVRRYT
   Ara h 1.01 (53-3) (442) ALMLPHFNSKAMVIVVVNKGTGNLELVAVRKEQQQRGRRE---EEEDEDEEEEGSNREVRRYT
   Ara h 1.01 (56-3) (442) ALMLPHFNSKAMVIVVVNKGTGNLELVAVRKEQQQRGRRE---EEEDEDEEEEGSNREVRRYT
   Ara h 1.01 (95-1) (442) ALMLPHFNSKAMVIVVVNKGTGNLELVAVRKEQQQRGRRE---EEEDEDEEEEGSNREVRRYT
     Ara h 1.02 (wt) (434) ALMLPHFNSKAMVIVVVNKGTGNLELVAVRKEQQQRGRREQEWEEEEEDEEEEGSNREVRRYT
    Ara h 1.02 (133) (102) ---------------------------------------------------------------
                                                                        Section 9
                (505) 505 510      520       530       540       550            567
     Ara h 1.01 (wt) (502) ARLKEGDVFIMPAAHPVAINASSELHLLGFGINAENNHRIFLAGDKDNVIDQIEKQAKDLAFP
   Ara h 1.01 (53-3) (502) ARLKEGDVFIMPAAHPVAINASSELHLLGFGINAENNHRIFLAGDKDNVIDQIEKQAKDLAFP
   Ara h 1.01 (56-3) (502) ARLKEGDVFIMPAAHPVAINASSELHLLGFGINAENNHRIFLAGDKDNVIDQIEKQAKDLAFP
   Ara h 1.01 (95-1) (502) ARLKEGDVFIMPAAHPVAINASSELHLLGFGINAENNHRIFLAGDKDNVIDQIEKQAKDLAFP
     Ara h 1.02 (wt) (497) ARLKEGDVFIMPAAHPVAINASSELHLLGFGINAENNHRIFLAGDKDNVIDQIEKQAKDLAFP
    Ara h 1.02 (133) (102) ---------------------------------------------------------------
                                                                       Section 10
                (568) 568         580       590       600       610             629
     Ara h 1.01 (wt) (565) GSGEQVEKLIKNQKESHFVSARPQSQSQSPSSPEKESPEKEDQEEENQGGKGPLLSILKAFN
   Ara h 1.01 (53-3) (565) GSGEQVEKLIKNQKESHFVSARPQSQSQSPSSPEKESPEKEDQEEENQGGKGPLLSILKAFN
   Ara h 1.01 (56-3) (565) GSGEQVEKLIKNQKESHFVSARPQSQSQSPSSPEKESPEKEDQEEENQGGKGPLLSILKAFN
   Ara h 1.01 (95-1) (565) GSGEQVEKLIKNQKESHFVSARPQSQSQSPSSPEKESPEKEDQEEENQGGKGPLLSILKAFN
     Ara h 1.02 (wt) (560) GSGEQVEKLIKNQRESHFVSARPQSQS--PSS-----PEKEDQEEENQGGKGPLLSILKAFN
    Ara h 1.02 (133) (102) --------------------------------------------------------------
```
