# Supplementary figures and images for: TILLING for allergen reduction and improvement of quality traits in peanut (Arachis hypogaea L.)
Source: BMC Plant Biol. 2011 May 12;11:81. doi: 10.1186/1471-2229-11-81 (PMC3113929; doi:10.1186/1471-2229-11-81)

**A.**

**Wild type (Tifrunner)**

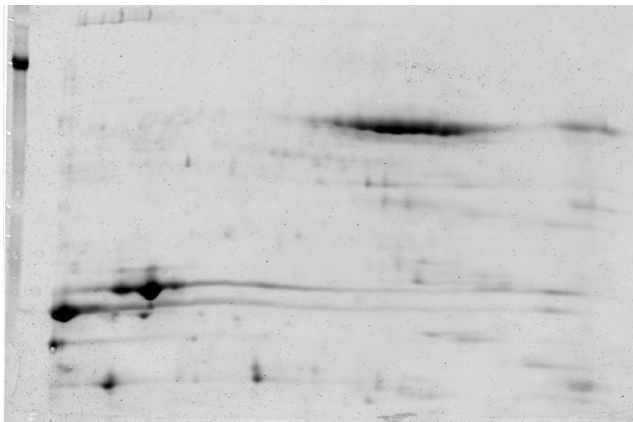

**Ara h 1.02 truncation mutant**

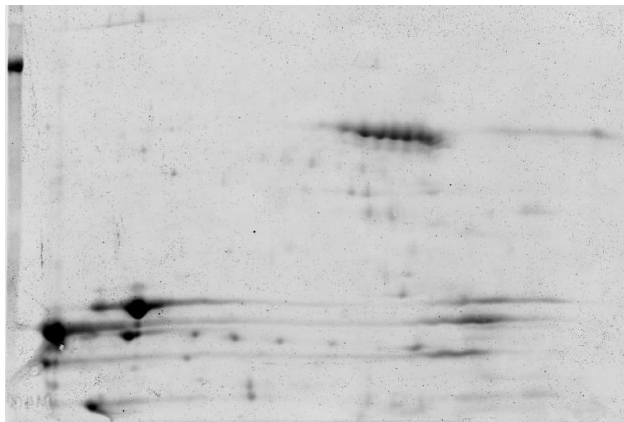

**B.**

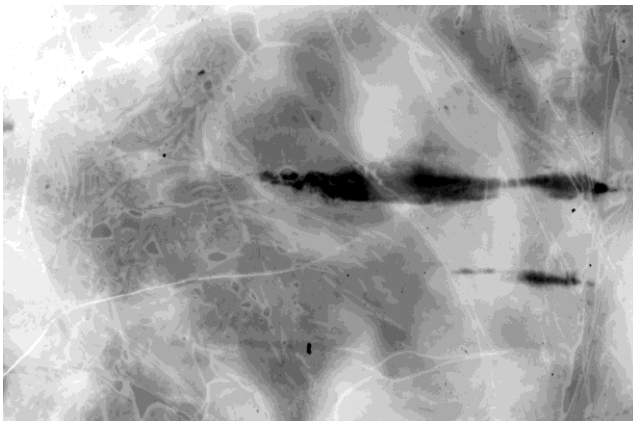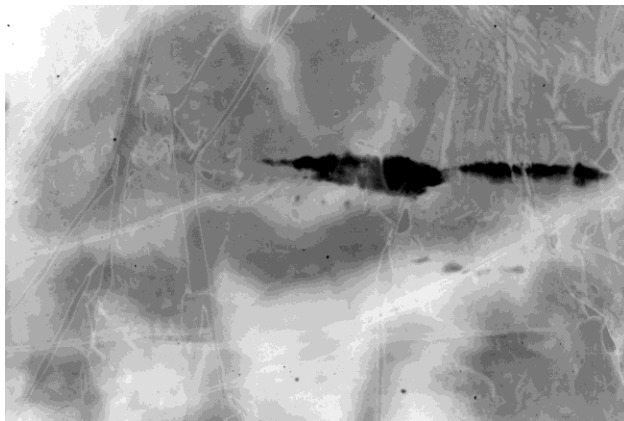

Supplement: Additional file 3 — 2D PAGE and Western blot of Ara h 1.02 truncation mutant. A - Sypro Ruby stained PVDF blots of seed protein extracts (1.5 mg) from wild-type (Tifrunner) and homozygous Ara h 1.02 truncation mutant. Proteins were first focused in pH 5.3 to 6.5 IPG strips, then separated in 10% polyacrylamide Tris-glycine gels before transblotting to PVDF membrane. B - Western blot of membranes in panel 7A using chicken anti-Ara h 1 antibody (primary) followed by anti-chicken-HRP conjugate (secondary), and visualized by fluorescence. [file 1471-2229-11-81-S3.PDF]
