# Supplementary material for: TILLING for allergen reduction and improvement of quality traits in peanut (Arachis hypogaea L.)
Source: BMC Plant Biol. 2011 May 12;11:81. doi: 10.1186/1471-2229-11-81 (PMC3113929; doi:10.1186/1471-2229-11-81)
Supplement: Additional file 4 — Sequence alignment of AhFAD2A and AhFAD2B wild-type proteins and predicted proteins from AhFAD2 mutants identified by TILLING. WT indicates wild-type protein sequence. Mutant ID numbers are indicated in parentheses. [file 1471-2229-11-81-S4.PDF]

```
                                                                     Section 1
                    (1)  1        10        20        30        40        50            64
    AhFAD2A (wt)    (1)  MGAGGRVTKIEAQKKPLSRVPHSNPPFSVGQLKKAIPPHCFERSLFISFSYVVYDLLVAYLLFY
    AhFAD2A (72-0)  (1)  MGAGGRVTKIEAQKKPLSRVPHSNPPFSVGQLKKAIPPHCFERSLFISFSYVVYDLLVAYLLFY
    AhFAD2A (4-3)   (1)  MGAGGRVTKIEAQKKPLSRVPHSNPPFSVGQLKKAIPPHCFERSLFISFSYVVYDLLVAYLLFY
    AhFAD2B (wt)    (1)  MGAGGRVTKIEAQKKPLSRVPHSNPPFSVGQLKKAIPPHCFERSLFISFSYVVYDLLMAYLLFY
    AhFAD2B (81-4)  (1)  MGAGGRVTKIEAQKKPLSRVPHSNPPFSVGQLKKAIPPHCFERSLFISFSYVVYDLLMAYLLFY
                                                                     Section 2
                   (65)  65   70        80        90        100       110            128
    AhFAD2A (wt)   (65)  IATTYFHKLPYPFSFLAWPIYWAIQGCILTGVWVIAHECGHHAFSKYQLVDDMVGLTLHSCLLV
    AhFAD2A (72-0) (65)  IATTYFHKLPYPFSFLAWPIYWAIQGCILTGVWVIAHECGHHAFSKYQLVDDMVGLTLHSCLLV
    AhFAD2A (4-3)  (65)  IATTYFHKLPYPFSFLAWPIYWAIQGCILTGVWVIAHECGHHAFSKYQLVDDMVGLTLHSCLLV
    AhFAD2B (wt)   (65)  IATTYFHKLPYPFSFLAWPIYWAIQGCILTGVWVIAHECGHHAFSKYQLVDDMVGLTLHSCLLV
    AhFAD2B (81-4) (65)  IATTYFHKLPYPFSFLAWPIYWAIQGCILTGVWVIAHECGHHAFSKYQLVDDMVGLTLHSCLLV
                                                                     Section 3
                  (129)  129       140       150       160       170       180         192
    AhFAD2A (wt)  (129)  PYFSWKISHRRHHSNTGSLDRNEVFVPKPKSKVSWYNKYMNNPPGRAISLFITLTLGWPLYLAF
    AhFAD2A (72-0)(129)  PYFSWKISHRRHHSNTGSLDRNEVFVPKPKSKVSWYNKYMNNPPGRAISLFITLTLGWPLYLAF
    AhFAD2A (4-3) (129)  PYFSWKISHRRHHSNTGSLDRDEVFVPKPKSKVSWYNKYMNNPPGRAISLFITLTLGWPLYLAF
    AhFAD2B (wt)  (129)  PYFSWKISHRRHHSNTGSLDRDEVFVPKPKSKVSWYNKYMNNPPGRAISLFITLTLGWPLYLAF
    AhFAD2B (81-4)(129)  PYFSWKISHRRHHSNTGSLRPRRSVCPETKIKGIMV----------------------------
                                                                     Section 4
                  (193)  193    200       210       220       230       240          256
    AhFAD2A (wt)  (193)  NVSGRPYDRFASHYDPYAPIYSNRERLLIYVSDSSVFAVTYLLYHIATLKGLGWVVCVYGVPLL
    AhFAD2A (72-0)(193)  NVSGRPYDRFASHYDPYAPIYSNRERLLIYVSDSSVFAVTYLLYHIATLKGLGWVVCVYGVLLL
    AhFAD2A (4-3) (193)  NVSGRPYDRFASHYDPYAPIYSNRERLLIYVSDSSVFAVTYLLYHIATLKGLGWVVCVYGVPLL
    AhFAD2B (wt)  (193)  NVSGRPYDRFASHYDPYAPIYSNRERLLIYVSDSSVFAVTYLLYHIATLKGLGWVVCVYGVPLL
    AhFAD2B (81-4)(165)  ----------------------------------------------------------------
                                                                     Section 5
                  (257)  257         270       280       290       300       310       320
    AhFAD2A (wt)  (257)  IVNGFLVTITYLQHTHASLPHYDSSEWDWLRGALATVDRDYGILNKAFHHITDTHVAHHLFSTM
    AhFAD2A (72-0)(257)  IVNGFLVTITYLQHTHASLPHYDSSEWDWLRGALATVDRDYGILNKAFHHITDTHVAHHLFSTM
    AhFAD2A (4-3) (257)  IVNGFLVTITYLQHTHASLPHYDSSEWDWLRGALATVDRDYGILNKAFHHITDTHVAHHLFSTM
    AhFAD2B (wt)  (257)  IVNGFLVTITYLQHTHASLPHYDSSEWDWLRGALATVDRDYGILNKAFHHITDTHVAHHLFSTM
    AhFAD2B (81-4)(165)  ----------------------------------------------------------------
                                                                     Section 6
                  (321)  321      330       340       350       360       370       380
    AhFAD2A (wt)  (321)  PHYHAMEATNAIKPILGDYYQFDGTPFYKALWREAKECLYVEPDDGASKKGVYWYKNKF-
    AhFAD2A (72-0)(321)  PHYHAMEATNAIKPILGDYYQFDGTPFYKALWREAKECLYVEPDDGASKKGVYWYKNKF-
    AhFAD2A (4-3) (321)  PHYHAMEATNAIKPILGDYYQFDGTPFYKALWREAKECLYVEPDDGASKKGVYWYKNKF-
    AhFAD2B (wt)  (321)  PHYRAMEATNAIKPILGDYYQFDGTPVYKALWREAKECLYVEPDDGASQKGVYWYKNKF-
    AhFAD2B (81-4)(165)  ------------------------------------------------------------
```
